# Enhanced Antioxidant, Antifungal, and Herbicidal Activities through Bioconversion of Diosgenin by *Yarrowia lipolytica P01a*

**DOI:** 10.3390/plants13182629

**Published:** 2024-09-20

**Authors:** Christian Hernández-Guzmán, Luis G. Hernández-Montiel, Adrian E. Velázquez-Lizarraga, Leopoldo J. Ríos-González, Sergio Huerta-Ochoa, Vianey de J. Cervantes-Güicho, Thelma K. Morales-Martínez, Claudio H. Mejía-Ruíz, Ana G. Reyes

**Affiliations:** 1 Unidad Iztapalapa, Departamento de Biotecnología, Universidad Autónoma Metropolitana, México City 02128, Mexico; christian_guzman24@hotmail.com (C.H.-G.);; 2 Centro de Investigaciones Biológicas del Noroeste, Programa de Agricultura en Zonas Áridas, Av. Instituto Politécnico Nacional 195, Playa Palo de Santa Rita Sur, La Paz 23096, Mexico; 3 Facultad de Ciencias Químicas, Universidad Autónoma de Coahuila, Saltillo, Coahuila 25280, Mexico

**Keywords:** saponins, bioherbicide, biofungicide, bioconversion with whole cells

## Abstract

This study explores the bioconversion of diosgenin by *Yarrowia lipolytica P01a*, focusing on enhancing the antioxidant, antifungal, and herbicidal activities of the resulting extracts. The bioconversion process, involving glycosylation and hydroxylation, produced significant amounts of protodioscin and soyasaponin I. The extracts showed superior antioxidant activity, with up to 97.02% inhibition of ABTS· radicals and 33.30% inhibition of DPPH· radicals at 1000 mg L^−1^ of diosgenin. Antifungal assays revealed strong inhibitory effects against *Botrytis cinerea*, *Alternaria* sp., and *Aspergillus niger*, with maximum inhibition rates of 67.34%, 35.63%, and 65.53%, respectively. Additionally, the herbicidal activity of the bioconverted extracts was comparable to commercial herbicides, achieving 100% inhibition of seed germination in both monocotyledonous and dicotyledonous plants. These findings suggest that the *Y. lipolytica P01a*-mediated bioconversion of diosgenin could provide a sustainable and eco-friendly alternative for developing natural biofungicides and bioherbicides.

## 1. Introduction

Saponins are a group of secondary metabolites produced by various plants, serving as part of their defense system against predators and pathogens [1]. These phytochemicals can be found in different plant parts, including barks, leaves, fruits, stems, roots, and seeds [2]. Saponins are characterized by their ability to produce foam, which has led to their traditional use as soaps [3]. Chemically, saponins consist of hydrophobic sapogenin (a steroid or triterpene aglycone) linked to hydrophilic sugar residues, resulting in molecules with amphiphilic properties [2].

These compounds have demonstrated a wide range of biological activities, including hemolytic, molluscicidal, anti-inflammatory, antiparasitic, antitumor, analgesic, antifungal, antibacterial, antiviral, allelopathic, and insecticidal properties [4,5,6,7,8,9,10,11,12]. Saponins are also important in the pharmaceutical industry as precursors for the synthesis of steroid hormone drugs [6,13]. Among these, diosgenin, a steroidal saponin, is particularly notable for its use in producing bioactive compounds with various therapeutic applications [14].

Saponins have garnered significant interest in the agricultural industry due to their potential as natural biopesticides and bioherbicides [15,16]. The excessive use of chemical pesticides since the 1960s has caused serious problems for human health and the environment [17]. The United States Environmental Protection Agency (EPA) has proposed the management of saponins in agriculture, approving them as safe biopesticides mainly for controlling fungi and viral diseases due to their molluscicidal, antiviral, and antifungal properties [17].

Quinoa saponins, for example, have been found to have antifungal activity against *Botrytis cinerea*, *Aspergillus niger*, and *Fusarium chlamydosporum* [18,19]. Additionally, they have shown toxicity against shrimp and molluscicidal activity against the apple snail (*Pomacea canaliculata Lamarck*), which affects rice crops [20]. Diseases caused by phytopathogenic microorganisms like bacteria, nematodes, and fungi result in substantial losses in agricultural production; thus, there is keen interest in the management of biopesticides, and saponins exhibit high potential as natural biofungicides [21].

Microbial bioconversion processes offer an alternative for the large-scale production of bioactive compounds involving biological systems that can chemically modify a precursor [22,23]. Common bioconversion reactions include hydroxylation and glycosylation to create new compounds with potential pharmacological and biological properties, making bioconversion a promising strategy for obtaining biologically active compounds [24,25]. The antioxidant activity of phytochemicals is linked to hydroxyl groups, and the increase in antioxidant activity is directly related to the degree of hydroxylation [22]. Previous research has demonstrated that these structural modifications can significantly improve bioactive compounds’ solubility, stability, and bioavailability, leading to enhanced pharmacological properties. For instance, using *Yarrowia lipolytica* in bioconversion processes has effectively modified steroidal saponins, improving their antioxidant and antimicrobial activities [26].

*Yarrowia lipolytica* is an oleaginous yeast known for its versatility in metabolizing a wide range of substrates and producing valuable metabolites, making it an attractive candidate for biotechnological applications [27]. It has been utilized for the bioconversion of various compounds, including steroids and saponins, to enhance their bioactivity and yield [22]. This yeast can express a variety of cytochrome P450 monooxygenases (CYP450s), which are crucial for hydroxylation reactions [27].

This study investigates the bioconversion of diosgenin by whole cells of *Yarrowia lipolytica P01a*, a marine-origin yeast, focusing on enhancing antioxidant, antifungal, and herbicidal activities in the resulting extracts. The bioconversion process primarily involves glycosylation and hydroxylation reactions, forming protodioscin and soyasaponin I, compounds with significant biological activities [22,25]. This study builds upon these findings by employing *Y. lipolytica P01a* to bioconvert diosgenin, a precursor of steroidal saponins, into glycosylated derivatives with potentially enhanced bioactivity. The findings of this research hold potential for developing natural biofungicides and bioherbicides, providing a safer and environmentally friendly alternative to synthetic chemicals in agriculture.

## 2. Results

### 2.1. Production of Protodioscin and Soyasaponin I

**Figure 1:**
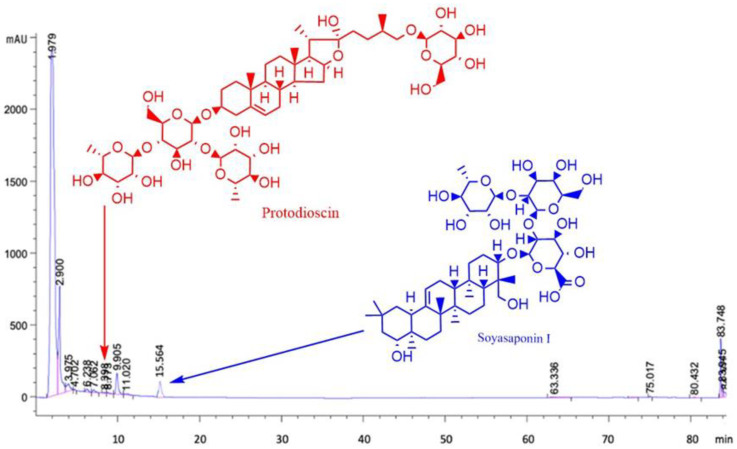
HPLC Analysis of Bioconverted Saponins by Yarrowia lipolytica P01a. The chromatogram shows the identification of protodioscin and soyasaponin I as the primary saponins produced during the bioconversion of diosgenin. Samples were analyzed at different initial concentrations of diosgenin (10–1000 mg/L). The chromatographic peaks correspond to retention times of known standards, confirming the successful bioconversion. Notably, no additional compounds were detected at higher diosgenin concentrations. This analysis highlights the glycosylation and hydroxylation modifications introduced by *Y. lipolytica* P01a, supporting the proposed bioconversion pathway.

**Figure 2:**
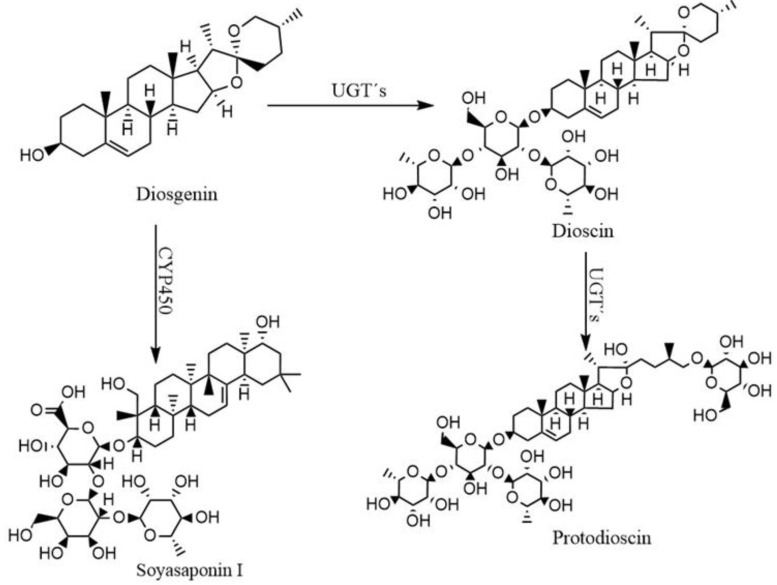
Proposed Metabolic Pathway for the Biosynthesis of Glycosylated Saponins by *Yarrowia lipolytica P01a*. The diagram illustrates the metabolic pathway for converting diosgenin into glycosylated steroidal saponins, including dioscin and protodioscin. The pathway highlights the critical role of UDP-glucosyltransferases (UGTs) in catalyzing the addition of glucose and rhamnose groups at the C-3 hydroxyl position. The pathway suggests potential enzymatic modifications involving cytochrome P450 monooxygenases (CYP450s), facilitating hydroxylation reactions. The diagram provides an overview of the bioconversion steps, producing bioactive saponins with pharmacological relevance.

The bioconversion process by *Y. lipolytica P01a* was carried out at different initial concentrations of diosgenin, which varied from 10 to 1000 mg L^−1^. The experimental part confirmed that the behavior of saponin production is different between concentrations. For example, two different compounds were present at low concentrations (10–40 mg L^−1^); only one compound was identified from 50 to 70 mg L^−1^ (Table 1). These compounds were identified by high-performance liquid chromatography (HPLC) (Figure 1). Regarding this study, the saponins protodioscin and soyasaponin I were biosynthesized by whole cells of *Y. lipolytica P01A* from diosgenin (Figure 2), which have not been previously reported as a product of this microbial bioconversion. It was not possible to relate the initial concentration of diosgenin with the bioconversion yield of the identified saponins (mg of products identified per mg of diosgenin consumed). This is because the synthesis of identified compounds decreased, and the formation of other unidentified compounds increased in a non-proportional relationship when the concentration of diosgenin increased. It was also observed that no residual diosgenin was present at all concentrations. This fact can be explained by the experiments that were performed using 100 mg L^−1^ of an initial concentration of diosgenin and *Y. lipolytica P01a* being previously inactivated by sterilization, presenting a recovery of 99.61% of the added diosgenin in the culture.

### 2.2. Antioxidant Activity

The bioconversion process was designed to enhance the antioxidant properties of diosgenin by introducing glycosyl and hydroxyl groups, which are known to increase radical scavenging activity. Antioxidant activity was measured using the ABTS· and DPPH· assays to evaluate this enhancement. The ABTS (2,2′-azino-bis(3-ethylbenzothiazoline-6-sulfonic acid)) and DPPH (1,1-diphenyl-2-picrylhydrazyl) assays are widely used to measure antioxidant activity by evaluating the radical-scavenging capacity of compounds. In the ABTS assay, the ABTS·+ radical, characterized by a blue-green color, is neutralized by antioxidants, causing a measurable reduction in absorbance at 734 nm. This method is versatile and suitable for both hydrophilic and lipophilic antioxidants. The DPPH assay involves reducing the purple DPPH· radical to DPPH-H, leading to a color change to yellow, which can be quantified by absorbance at 517 nm. This method is commonly used for evaluating antioxidants in organic solvents. In this study, the bioconverted diosgenin extracts exhibited strong antioxidant activity in both assays, with ABTS showing a higher sensitivity. The improved radical scavenging activity is attributed to the structural modifications introduced during the bioconversion process, which enhanced the antioxidant potential of the extracts. The goal was to assess whether the bioconverted extracts showed improved antioxidant potential compared to the unmodified diosgenin.

The experimental setup involved testing extracts obtained from different initial concentrations of diosgenin (ranging from 10 to 1000 mg/L) and comparing their radical scavenging activity against ABTS· and DPPH· radicals. The results demonstrated that the bioconversion significantly boosted antioxidant activity. For instance, the extract derived from 1000 mg/L of diosgenin showed a 97.02% inhibition of ABTS· radicals and 33.30% inhibition of DPPH· radicals. In contrast, the unconverted diosgenin exhibited substantially lower inhibition rates.

Interestingly, extracts obtained from lower initial concentrations of diosgenin (e.g., 10 mg/L) displayed a 6.62-fold higher scavenging activity against ABTS· radicals and a 16.59-fold increase in DPPH· radical inhibition compared to the precursor molecule. These results highlight the enhanced antioxidant potential of the bioconverted extracts, which can be attributed to the formation of protodioscin and soyasaponin I during the bioconversion process.

**Figure 3:**
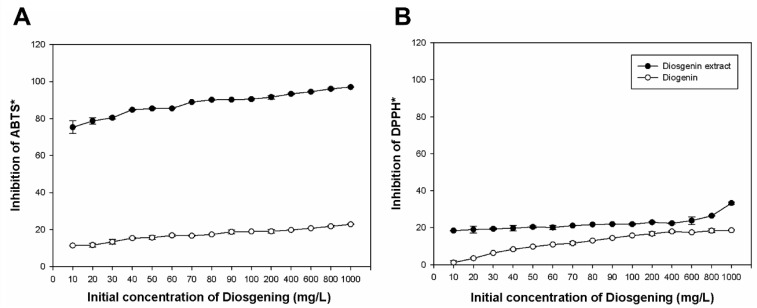
Antioxidant Activity of Bioconverted Diosgenin Extracts. The bar graphs depict the antioxidant activity of the bioconverted diosgenin extracts, measured as the percentage of inhibition of ABTS· (**A**), and DPPH· (**B**) radicals. Extracts were derived from different initial diosgenin concentrations (ranging from 10 to 1000 mg/L). The results show a significant increase in radical scavenging activity compared to the unmodified diosgenin. The highest levels of antioxidant activity were observed at higher diosgenin concentrations, indicating the enhanced efficacy of the bioconversion process in generating bioactive saponins. Error bars represent standard deviations from triplicate experiments. * *Means radicals*.

Figure 3 illustrates these findings, showing the comparative inhibition percentages for both ABTS· and DPPH· radicals across various concentrations of diosgenin. The data indicate that bioconversion by Yarrowia lipolytica P01a effectively improves the antioxidant capacity of diosgenin, making these extracts promising candidates for applications requiring strong antioxidant activity.

### 2.3. Antifungal Activity

Given the potential of saponins as natural antifungal agents, this section aimed to determine whether the bioconversion of diosgenin by *Yarrowia lipolytica P01a* could enhance antifungal activity against key agricultural pathogens. Specifically, the extracts were tested against *Botrytis cinerea*, *Alternaria* sp., *and Aspergillus niger* to evaluate their inhibitory effects.

The antifungal activity was assessed using a radial growth inhibition assay on agar plates, where extracts from the bioconversion process were introduced at various concentrations. The results demonstrated that *Botrytis cinerea* was the most sensitive to the bioconverted extracts, showing up to 67.34% inhibition at certain diosgenin concentrations. In contrast, the inhibition of *Alternaria* sp. and *Aspergillus niger* varied depending on the initial diosgenin concentration and the duration of exposure, with maximum inhibition rates of 35.63% and 65.53%, respectively. The detailed percentages of inhibition for each fungal species are presented in the supplementary tables (Tables S1–S3). These tables provide comprehensive data on the impact of the extracts, including comparisons between the untreated control and treated samples.

**Figure 4:**
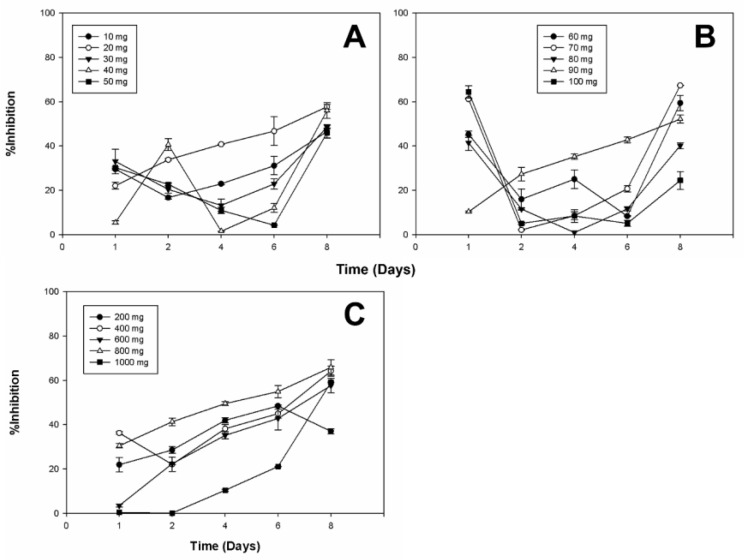
Inhibition of *Botrytis* cinerea Growth by Bioconverted Diosgenin Extracts. The bar chart illustrates the inhibitory effects of the bioconverted extracts on the radial growth of *Botrytis cinerea*. Inhibition percentages are presented for different initial concentrations of diosgenin used in the bioconversion process: (**A**) from 10 to 50 mg; (**B**) from 60 to 100 mg; (**C**) from 200 to 1000 mg. The data indicate a dose-dependent inhibition, with maximum growth inhibition observed at higher diosgenin concentrations. Controls included untreated fungal cultures and cultures treated with non-bioconverted diosgenin (not included in the figure because it did not affect the fungal strains). This figure underscores the potential of bioconverted diosgenin extracts as effective antifungal agents.

**Figure 5:**
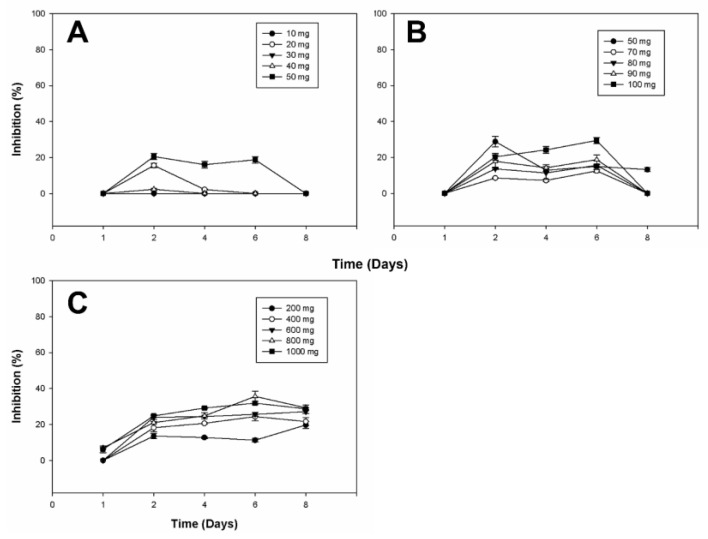
Inhibition of *Alternaria* sp. Growth by Bioconverted Diosgenin Extracts. This figure presents the effects of the bioconverted diosgenin extracts on the radial growth inhibition of *Alternaria* sp. The graph shows inhibition percentages across different initial diosgenin concentrations: (**A**) from 10 to 50 mg; (**B**) from 60 to 100 mg; (**C**) from 200 to 1000 mg. The extracts exhibited strong antifungal activity, particularly at higher concentrations, where the inhibition rates approached those seen with commercial antifungal agents. The controls included untreated cultures and cultures treated with unmodified diosgenin (not included in the figure because it did not affect the fungal strains). These results suggest that bioconverted diosgenin may serve as a viable natural fungicide.

**Figure 6:**
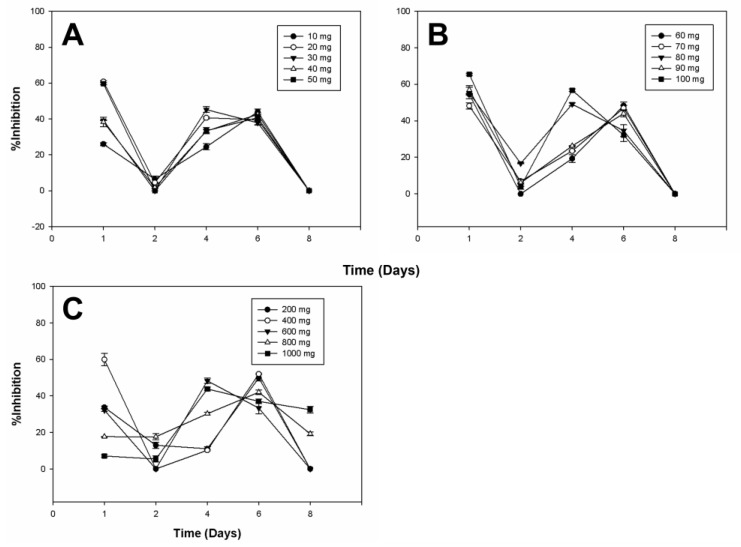
Inhibition of *Aspergillus niger* Growth by Bioconverted Diosgenin Extracts. The graph illustrates the percentage inhibition of *Aspergillus niger* growth by extracts obtained from the bioconversion of diosgenin at varying initial concentrations: (**A**) from 10 to 50 mg; (**B**) from 60 to 100 mg; (**C**) from 200 to 1000 mg. Although the inhibition observed was lower than that of other fungi, significant antifungal effects were still noted, particularly at mid-range concentrations. This moderate inhibitory effect highlights the differential sensitivity of fungal species to the bioconverted extracts. The controls included untreated cultures and cultures treated with unmodified diosgenin (not included in the figure because it did not affect the fungal strains). The results reinforce the importance of optimizing extract concentrations for targeted antifungal applications.

Figure 4, Figure 5 and Figure 6 provide a detailed overview of the antifungal activity observed across different extracts. We selected specific diosgenin concentration ranges (e.g., 20 mg/L and 60–80 mg/L) based on our preliminary experiments that indicated these points as critical for capturing non-linear effects in bioactivity. These ranges allow for an in-depth analysis of the relationship between substrate concentration and bioconversion efficiency, revealing the threshold effects and saturation points crucial for optimizing product yield and efficacy. The data highlight the efficiency depending on the microorganism and the diosgenin concentration used in bioconversion. The observed non-linear trends in the inhibition of fungal growth across different diosgenin concentrations highlight the complex dynamics of the bioconversion process. As the diosgenin concentration increases, enzyme saturation and substrate availability play significant roles in determining the final bioactivity of the extracts. These findings underscore the potential of using bioconverted diosgenin extracts as eco-friendly alternatives for controlling fungal pathogens in agricultural settings.

The effectiveness of the extracts suggests a synergistic interaction between the saponins produced during bioconversion. Notably, the bioconverted extracts achieved levels of inhibition comparable to some commercial fungicides. Figure 6 illustrates that while the bioconverted diosgenin extracts inhibited the growth of *Aspergillus niger*, the degree of inhibition varied across different concentrations and time points. Notably, the inhibition was more pronounced at certain times, such as on Day 1 and Day 6, indicating a variable response by *A. niger* to the bioactive compounds.

Considering that the tests showed antifungal activity at different times, the activity on microorganisms may be due to a single active principle or the synergistic action of several of them found in the extracts. There are references to using plant extracts as antifungals on various microorganisms. Still, according to the literature, no data is available for extracts from bioconversion processes with whole cells, much less with *Y. lipolytica P01a*. The inhibition of different microorganisms through extracts from the bioconversion process can produce new biofungicides from other saponins, such as the precursor diosgenin and whole cells.

### 2.4. Herbicidal Activity

This section investigates whether the bioconverted diosgenin extracts exhibit herbicidal properties comparable to commercial herbicides like Picloram+2,4-D, used as a control in this study. Considering the known phytotoxic effects of saponins, this study evaluated the impact of these extracts on the germination of various monocotyledonous and dicotyledonous seeds, including corn, wheat, barley, and beans (Table 2).

The herbicidal activity was assessed by measuring the inhibition of seed germination in controlled environmental conditions. The results revealed that the bioconverted extracts effectively prevented seed germination across all tested species. Notably, 100% inhibition was observed at multiple initial diosgenin concentrations, indicating that the bioconverted extracts possess herbicidal solid properties. This effect was consistent for monocotyledonous and dicotyledonous plants, matching the performance of the commercial herbicide used as a control. Detailed visual evidence of the herbicidal effects is available in Supplementary Figures S1 and S2, which depict the inhibitory effects on seed germination of maize and bean, respectively, compared to the controls, which were the *Yarrowia lipolytica P01a* extract and the standard herbicides.

The data also showed that even at lower concentrations (e.g., 10–20 mg/L), the bioconverted extracts significantly reduced germination rates, suggesting a dose-dependent response. The inhibition patterns indicate that the bioconversion process enhances the phytotoxicity of diosgenin, likely due to the formation of saponins with herbicidal activity, such as protodioscin and soyasaponin I.

Table 2 summarizes these findings, comparing the herbicidal effects of the bioconverted extracts, pure diosgenin, and commercial herbicide. The results highlight the potential of these bioconverted extracts as environmentally friendly alternatives for weed management in agricultural systems, offering a sustainable option for controlling invasive species while reducing reliance on synthetic chemicals.

## 3. Discussion

The HPLC analysis of saponins produced by *Y. lipolytica P01a* in liquid culture revealed the presence of protodioscin and soyasaponin I (Figure 1, Table 1), while at high concentrations, no compounds were determined. Bioconversion reactions by *Y. lipolytica P01a* include glycosylation and hydroxylation reactions as the main modifications to the native diosgenin molecule. This fact agrees with the study by Hernández-Guzmán et al., [22] where they performed the bioconversion of naringenin using whole cells of *Yarrowia lipolytica 2.2ab* as a biocatalyst, obtaining hydroxylated compounds with high added value and a compound with a molecular weight of 475 g mol^−1^, which could not be compared with the library of the computer software (UPLC-MS/MS) but indicated that the new compound was a glycosylated phytochemical. The bioconversion of diosgenin by *Yarrowia lipolytica P01a* involves a complex network of enzymatic reactions, primarily catalyzed by cytochrome P450 monooxygenases (CYP450s) and UDP-glycosyltransferases (UGTs). The *Y. lipolytica* genome encodes approximately 12 CYP450 enzymes and many UGTs, both of which are essential for the hydroxylation and glycosylation steps, respectively, required for the synthesis of glycosylated saponins such as Soyasaponin I [28,29,30]. Understanding the specific localization of these reactions within the cell can provide deeper insights into the efficiency and outcome of the bioconversion process. While CYP450 enzymes are typically localized in the endoplasmic reticulum (ER), where they facilitate hydroxylation reactions, UGTs are thought to operate in the cytosol or on the cytoplasmic side of the ER membrane, where they catalyze the glycosylation of hydroxylated intermediates [31]. These cellular compartments likely play critical roles in the import, conversion, and eventual export of the bioconverted saponins. The precise localization and pathway dynamics remain areas for further research but can be useful for optimizing the production of bioactive saponins in industrial applications. Hydroxylation is the reaction mechanism that stands out in the microbial bioconversion of saponins [24,25,32]. For the biosynthesis of glycosylated steroidal saponins such as protodioscin, the specific metabolic pathway for *Y. lipolytica P01a* is proposed in this study, starting from diosgenin as a precursor (Figure 2). The biosynthesis of dioscin and protosioscin, as well as the other derivatives, requires the addition of glucose groups and rhamnose groups in the C-3 hydroxyl, which is catalyzed by the enzymes called UDP-glucosyl transferase (UGT’s) [33].

Likewise, the dioscin-derived saponin was not identified and quantified in the extracts from the bioconversion. Soyasaponin I belongs to the group of triterpene saponins as a hydroxylated, glycosylated, and methylated form of diosgenin biocatalyzed by *Y. lipolytica P01a*. The production of soyasaponin was a maximum of 18.04 ± 0.01 mg L^−1^ using 70 mg L^−1^ of initial diosgenin; so far, there have been no reports promoting the bioconversion of diosgenin to soyasaponin I by biological systems such as biocatalysts. *Yarrowia lipolytica* has been shown to express the enzyme cytochrome P450 monooxygenase (CYP450), capable of catalyzing reactions of hydroxylation, reduction, formation of C=C double bonds, and glycosylation of different bioactive compounds [22]. Notably, at an initial concentration of 20 mg L^−1^ of diosgenin, the identified products were present with a yield (Yp/s) of 0.969, the highest of the thirteen concentrations tested. The amount of bioactive saponins produced was 19.39 mg, obtaining 0.969 mg of product per mg of diosgenin. This result is relevant considering that saponins possess various pharmacological properties, such as regulating immune function, improving the cardiovascular system, and exhibiting analgesic, antitumor, and anti-inflammatory activities [1,2,7,8]. Coupled with this, they can be used clinically to treat thrombosis, asthma, tumors, arthritis, and coronary disease. They are also of great interest in the pharmaceutical industry because they are used as raw materials for synthesizing steroid hormone drugs. In the agricultural industry, saponins, due to their properties, are used as a biocontroller or biopesticide for phytopathogenic fungi. [13,18,34,35]. In comparing the results of our study with those of previous bioconversion processes, it becomes evident that using *Yarrowia lipolytica P01a* as a biocatalyst offers a competitive advantage in terms of saponin yield and enhanced bioactivity. Table 3 provides a comprehensive comparison of saponin production through various microbial systems, highlighting the unique contributions of each method.

Our findings demonstrate that the bioconverted diosgenin produced up to 19.39 mg/L of saponins, specifically protodioscin and soyasaponin I, which exhibit significant antioxidant and antifungal activities. This yield is comparable to or exceeds those reported in studies utilizing other microbial systems, such as *Cunninghamella blakesleeana*, *Penicillium dioscin*, and *Aspergillus niger.* Notably, the enhanced bioactivity observed in our study underscores the potential of *Yarrowia lipolytica P01a* for producing high-value saponins with applications in pharmaceuticals and agriculture.

The comparative analysis in Table 3 highlights that while various microbial systems can transform diosgenin into bioactive saponins, the efficiency and specificity of the modifications achieved in our study suggest that *Yarrowia lipolytica P01a* is particularly effective for this purpose. This result validates the approach and suggests potential areas for further optimization and application in industrial processes.

In the agricultural sector, the biocidal effect of saponins has been evaluated [21]. There is evidence of the antifungal activity of an extract rich in saponins that came from waste from the industrial process of *Chenopodium quinoa Willd* (quinoa) grains, finding good activity of the extract against the fungus *Cercospora beticola*, a pest that affects chard crops. Chemical pesticides have generated benefits in agricultural production. However, their inappropriate use has contaminated the soil, water, air, and agricultural products; this is due to the accumulation of residues potentially harmful to human health, animals, and the environment, interpreted in terms of type, toxicity, number of applications and doses of these chemical pesticides [42]. Thus, interest has increased considerably in the use of plant extracts with promising results in agriculture demonstrating controlling effects against mites, rodents, nematodes, bacteria, viruses, fungi, and insects, using plant species from various environments ecological and abundant in nature [43,44]. In addition to this, the juice extracted from the fique, which has saponins, has been used as a biofungicide against the phytopathogenic fungus *Colletotrichum gloeosporioides*, which is responsible for the presence of necrotic spots (anthracnose) on leaves, young branches, inflorescences, and fruits of the Tamarillo fruit, demonstrating good antifungal activity [44]. Hata Yoshie et al. [45] used different varieties of Dioscorea spp. to quantify sapogenins and the species studied. The most promising for sapogenin content is *Dioscorea rotundata*, with an average of 28.4 mg 100 g^−1^ of tuber on a dry basis, and 34% of the samples analyzed contained diosgenin. In another related study, Navarro del Hierro et al. [45] reported the extraction of saponins from edible seeds (quinoa, soybean, red lentil, fenugreek, and lupine) assisted by ultrasound, demonstrating that fenugreek showed the highest total saponin content (mean value 95 g 100 g^−1^).

In comparison, soybean and lupine showed the lowest values (mean value around 3 g 100 g^−1^). Lentil and quinoa showed intermediate contents of total saponins (mean value around 6 g 100 g^−1^ and 4 g 100 g^−1^, respectively). Puente-Garza et al. [46] quantified glycosylated saponins through protodioscin equivalents (mg Protodioscin/g sample) in various agave species, obtaining between 43.4 and 66 mg Protodioscin/g of sample. This report obtained a higher production of saponins than in previous studies. Therefore, this work demonstrates that bioconversion can overcome the extraction restrictions from plant sources of this type of compound with a high added value. It makes them attractive candidates for use as biopesticides and bioherbicides of interest for various industries such as agriculture.

Different phytochemicals, such as saponins from secondary metabolism in plants, have been shown to influence various biological activities that can benefit animal and human health and the environment [47]. For this reason, the antioxidant capacity of the extracts obtained from the bioconversion process of saponin diosgenin was measured. Osadebe et al. [48] reported percentages of inhibition of vitamin C, considered one of the compounds with excellent antioxidant potential. They obtained percentages of inhibition of 92.8 to 98% of the DPPH radical using 2500 to 3000 mg L^−1^ of vitamin C. Saponins extracted from various plants have been shown to have various bioactive effects. Among these, anti-inflammatory, antifungal, antimicrobial, antiparasitic, and antioxidant activity stands out [1,2,6,9,49]. As can be seen in Figure 3, the percentage of inhibition of the ABTS· radical is greater than that of the DPPH· radical, indicating that there is a significant difference between these percentages of inhibition of the synthetic radicals ABTS· and DPPH· in the extracts obtained of the bioconversion process. In addition, there is reversibility in the reaction of the DPPH· radical with compounds such as phytochemicals, which would cause a decrease in antioxidant activity, thus clarifying the disparity between the data obtained [50]. On the other hand, the difference between the percentage of inhibition of ABTS· and DPPH· radicals is because most plant compounds have better antioxidant activity against ABTS· radicals than DPPH· radicals. Because there is an increase in the sensitivity of the ABTS assay to identify antioxidant activity, the kinetic reaction is expected to be faster, attributing greater antioxidant activity to the ABTS method [51]. Chau Miranda et al. [52] aimed to evaluate the in vitro antioxidant activity of the different extracts obtained from the fruit of *Physalis peruviana* L. (aguaymanto) and fresh fruit using the DPPH·, ABTS· method; saponins were identified among the phytochemical components. They stated that the lyophilized aqueous extract has greater antioxidant capacity by both methods (DPPH·: 20.55 μg/mL) and (ABTS·: 2.48 ± 0.04 μmol trolox/g sample) compared to the aqueous extract and fresh fruit. González et al. [47] carried out the total antioxidant activity of ethanolic extracts of leaves, young stems, and roots of *Tithonia diversifolia*. Presenting greater antioxidant capacity in the root extract, in which saponins were identified. *T. diversifolia* presented good antioxidant activity when tested in DPPH· and ferric reduction assays, and the species of the *Asteraceae* family stood out for demonstrating high values of antioxidant activity against the DPPH· radical [53,54]. A study by Cerna et al. [55] identified secondary metabolites in ethanolic extracts of 35 species of orchid genera through a phytochemical screening, where tests were carried out for different phytochemicals, including saponins. They evaluated the antioxidant capacity of the various species of orchids using the DPPH· method to analyze the free radical scavenger capacity. The most notable species were *Oncidium excavatum* and *Epidendrum nocturnun*; the latter species stood out for requiring a concentration of 3.50 ppm to inhibit 50% of the DPPH· free radicals present in the test solution. In another similar study, the presence of secondary metabolites in the culms of *Guadua angustifolia Kunth* was evaluated, and their antioxidant activity was determined. In these samples, phytochemical compounds such as saponins, among others, were detected. The DPPH· and ABTS· methods also measured the antioxidant activity, producing high activity in ethanolic extracts [56]. In this study, we observed that all bioconverted extracts, except the unmodified diosgenin, contained a variety of structurally modified compounds. Specifically, the bioconversion process introduced glycosylation and hydroxylation modifications, which influence the antioxidant activity of phytochemicals. Our findings demonstrated that these structural modifications significantly enhanced the antioxidant capacity of the biosynthesized compounds compared to the original diosgenin. This enhancement can be attributed to the increased solubility and stability of the modified saponins, consistent with previous studies showing similar bioactivity improvements following glycosylation [22]. However, our work goes further by identifying specific bioconverted products and correlating their structural changes directly with enhanced antioxidant activity. This approach confirms the general principle that structural modifications can alter bioactivity and provides detailed insights into the specific mechanisms at play in the bioconversion of diosgenin by *Yarrowia lipolytica P01a.*

The diosgenin extracts from the bioconversion process showed an inhibitory effect at different times on the growth of three other fungi: *Botrytis cinerea*, *Alternaria* sp., and *Aspergillus niger*. The highest inhibition of *Botrytis cinerea*, *Alternaria* sp., and *Aspergillus niger* was 67.34 ± 0.48%, 35.63 ± 2.82%, and 65.53 ± 0.53%, with the extracts of 70 mg L^−1^, 800 mg L^−1^ and 100 mg L^−1^ of initial diosgenin, and at 8, 6 and 1 days of treatment, respectively (Supplementary Data). The variable inhibition of *Aspergillus niger* observed in Figure 6 suggests that while the bioconverted diosgenin extracts do exert an inhibitory effect, the extent of this effect is influenced by factors such as the concentration of active compounds, their stability over time, and the inherent resistance mechanisms of *A. niger*. This finding highlights the need for further optimization of the bioconversion process to enhance the efficacy of the extracts against more resistant fungal species. Different authors have reported on the antifungal activity of various plant extracts containing different bioactive compounds, such as saponins used against *Botrytis cinerea* [19,57], *Alternaria* sp. [48], and *Aspergillus* [58]. Ramírez-Salcedo et al. [59] carried out the inhibition of *Botrytis cinerea* with extracts of *Guazuma ulmifolia*, obtaining the highest concentration of 85.5 ± 2.1% with 10 mg mL^−1^ of fruit extract and 13 days of treatment. They also compared these data with a commercial fungicide called benomyl and found an inhibitory effect like the commercial fungicide of 91.5 ± 1.6% using a concentration of 1 mg mL^−1^ of benomyl. The mycelial growth of *A. alternata* was inhibited by chiltepin phenolic extracts after the third and fifth day of growth, obtaining 41.28% and 38.46% inhibition, respectively, suggesting that phenolic compounds interact with the cell wall of the fungus and thus inhibit some fungal enzymes to prevent mycelium growth from being achieved [60]. Quiroga et al. [58] reported the use of alcoholic extracts of the plant called pirúl (*Schinus molle* L.), which presented inhibition in the growth of *A. niger.*

The herbicidal activity of the bioconverted diosgenin extracts was assessed on both monocotyledonous (*Zea mays* L., *Triticum aestivum*, *Hordeum vulgare*) and dicotyledonous (*Phaseolus vulgaris*) model seeds. The results demonstrated significant herbicidal inhibition across all tested species. The extracts effectively inhibited seed germination, indicating their potential as natural herbicides derived from bioconversion. These findings underscore the versatility of bioconverted diosgenin as an eco-friendly alternative for controlling weed growth in agricultural settings. The detailed results of the herbicidal inhibition are summarized in Table 2, which provides a comparative analysis of the inhibition percentages across the different seed types. Weeds are a notable risk to the integrity of natural and agricultural environments due to their invasive and competitive potential [61]. Herbicides are generally all toxic and pollute the environment, so there is a constant search for environmentally friendly control alternatives to combat weeds. In this regard, bioactive compounds or secondary metabolites from plants or microorganisms with properties of limiting seed germination and reducing growth are usable in agriculture. Thus, they represent an alternative to new practices that reduce environmental impact and preserve the cell structure of crops. Plant or microorganism´ phytotoxic compounds are potential candidates for bioherbicides [47,62]. Several studies have reported saponins’ allelopathic or phytotoxic properties, suggesting they could be evaluated as potential bioherbicides [63,64]. At low initial concentrations of the diosgenin control of 10–30 mg L^−1^, in the case of monocotyledonous seeds and 10–20 mg L^−1^ in dicotyledonous seeds, there was a significant difference compared to the bioconversion and herbicide extracts (*p* ≤ 0.05). It should be noted that diosgenin has herbicidal activity in all initial concentrations, reaching 100% inhibition in monocotyledonous seeds from 40 mg L^−1^ and in dicotyledonous seeds from 30 mg L^−1^. However, after the bioconversion process, it obtained a 100% inhibitory effect on the germination of the various seeds tested. There are multiple reports where extracts from different plant sources are used, showing unfavorable effects on germination in numerous crops, such as the use of methanolic extracts from radish (*Raphanus sativus*) and rutabaga (*Brassica napobrassica*), which presented an inhibitory effect on germination in lettuce (*Lactuca sativa*), tomato (*Solanum lycopersicum*) and rice (*Oryza sativa*) crops [65]. The allelopathic effect of phytochemicals (flavonoids, alkaloids, terpenoids, and saponins) from the plant’s *Camellia sinensis*, *Zanthoxylum armatum*, *Amomum subulatum*, *Eupatorium glandulosum*, *Cymbopogan flexuosus*, and *Rhododendron arboreum* on germination, radicle length, and root length has been evaluated of the plumule of bean and radish seeds, concluding that these plants have a large number of secondary metabolites that inhibited the development of bean and radish seeds so that they grew vigorously. Therefore, these plants could be a wonderful source of herbicides to suppress the growth of unwanted weeds in the field [66]. Findings have also been demonstrated suggesting that cassava leaves show the presence of several phytochemical groups, including saponins, tannins, steroids, alkaloids, phenolics, and flavonoids, which can be used as new herbicidal agents for the control of weeds such as *Eleusine indica*, *Ageratum conyzoides*, *Cyperus distans* in aerobic rice cultivation [67]. González et al. [47] evaluated the herbicidal effect of the vegetable water extract and a commercial herbicide (active principle: Simazine). Evaluating through wheat seed germination tests, they tested the concentration of total phenols of 480 ppm, where there was no germination of the seeds. Saponins are among the phytochemicals often considered to have direct or indirect effects and can be used under natural conditions due to their water solubility. The germination of various seeds can be negatively affected by saponins in extracts from different plants. These bioactive compounds act as inhibitors of mixed enzymatic reactions and can also exert phytotoxicity through their effects on seed membrane lipids in some seed species [68,69]. The impact of the aqueous extract of total saponins of *Quillaja lancifolia* at 4 and 10% (*w*/*v*) on the morphophysiological parameters of *Lactuca sativa* (lettuce) and *Echinochloa crus-galli* (barn grass) has been examined in pre- and post-emergence bioassays, presenting a significant decrease of more than ten times the growth of germinated lettuce seedlings and phytotoxic effects were also observed on the growth of barnyard grass [61]. Undesirable results have been obtained due to the intensive use of chemical herbicides; the consequences are the contamination of soil and wastewater, accumulation of their residues in crops, and the emergence of weed populations resistant to chemical herbicides. Based on this, the development of environmentally friendly herbicides with new action mechanisms is notable. Among the compounds used in herbicide formulations are natural phytotoxins of plant or microbial origin [70]. Due to those mentioned earlier, this study aims to show the advances in developing new biological bioherbicides with high phytotoxic potential from diosgenin extracts from bioconversion processes.

## 4. Materials and Methods

### 4.1. Microorganism

*Yarrowia lipolytica P01a* was the strain employed throughout this experimental study. It was obtained from the collection of the Biotechnology Department of the Centro de Investigaciones Biologicas del Noroeste Campus La Paz, Mexico. The fungi used to carry out the antifungal activity were obtained from the collection of the environmental biotechnology laboratory of the Autonomous University of Coahuila campus in Saltillo, Mexico. The strains were propagated in Erlenmeyer flasks with Potato Dextrose Agar medium (PDA) and incubated for 7 days at 30 °C [22].

### 4.2. Chemicals

Diosgenin (CAS 512-04-9), protodioscin (CAS 51330-27-9), soyasaponin (CAS 114590-20-4), and free radicals DPPH· (1,1-Diphenyl-2-picrylhydrazyl) and ABTS· were purchased from Sigma-Aldrich (St. Louis, MA, USA). Potato Dextrose Agar Medium (PDA) and Sabouraud Dextrose Broth (SDB) were supplied by Bioxon (Franklin Lakes, NJ, USA). Distilled water was used for all experiments.

### 4.3. Whole-Cell Bioconversion

Before bioconversion, *Y. lipolytica P01a* was incubated in 250 mL Erlenmeyer flasks with 100 mL of broth SDB medium with 150 μL of inoculum equivalent to 1 × 10^6^ cells mL^−1^ at 150 rpm for 24 h at 30 °C. After the cell growth process, the diosgenin was added to initiate the bioconversion. Thirteen initial concentrations of diosgenin, 10, 20, 30, 40, 50, 60, 70, 80, 90, 100, 200,400, 600, 800, and 1000 mg L^−1^ were tested. Cultures were incubated for 8 days at 30 °C. The control was diosgenin in a sterile medium without microorganisms and microorganisms previously inactivated by sterilization, as well as a sterile medium with active microorganisms and without diosgenin. Each experiment was performed in triplicate [22].

### 4.4. Sample Preparation

The bioconversion process samples of 5 mL from the supernatant were obtained after 192 h of bioconversion. Samples were filtered, thus separating the supernatant from the cells, and extracted the phytochemicals from the supernatant by ultrasound-assisted extraction (UAE) homogenized with 5 mL of methanol/water (60/40, *v*/*v*). The UAE was performed for 45 min at 80.0 Hz and 40 °C; the extracts (10 mL) were filtered at 0.22 μm (Whatman Uniflow Syringe Filters, Cytiva, Marlborough, MA, USA) and concentrated at 60 °C using a vacuum rotary evaporator. The extracts were solubilized in 2 mL of methanol and stored at 4 °C.

### 4.5. Product Analysis by HPLC-UV

The determination of saponins in the extracts was determined using an Agilent 1260 Infinity HPLC equipment (Agilent Technologies, Santa Clara, CA, USA), with a diode array detector (UV–VIS) (Agilent Technologies, Santa Clara, CA, USA) and an Agilent Zorbax C18 ODS (Agilent Technologies, Santa Clara, CA, USA) column (250 mm × 4.6 mm) at a temperature of 35 °C. It was used with acetonitrile (phase A) and 80% water (phase B) in the mobile phase with a 1 mL min^−1^ flow rate. The injection volume was 20 μL in a gradient elution Phase A linear gradient from 20 to 30% for 5 min, linear gradient from phase A of 30 to 40% for 30 min, linear gradient of phase A from 40 to 60% for 5 min, linear gradient of phase A from 60 to 85% for 20 min, linear gradient of phase A from 85 to 100% for 15 min, an isocratic elution of phase A of 100% for 5 min, and an linear gradient of phase A from 100 to 20% for 5 min. Commercial standards of diosgenin, protodioscin, and soyasaponin were used for identification according to their characteristic retention times, which were visualized and analyzed using the Agilent OpenLAB software v2.8. Once the products were quantified, the total yield of bioconversion of saponins was determined by the milligrams of total products per milligram of diosgenin consumed.

### 4.6. Determination of Antioxidant Activity

Antioxidant activity was measured by the ABTS· and DPPH· free radical scavenging methods. The ABTS· radical elimination test was conducted with a solution of 7 mM of ABTS· and 2.45 mM of a potassium persulfate solution. These solutions were mixed in equal parts and kept in the dark at room temperature for 12 to 16 h to form the ABTS· radical. After 12 to 16 h, the ABTS· solution was diluted in ethanol to absorb 0.8 (±0.02) at 734 nm, and the blank used was ethanol. For sample analysis, 100 μL of the extract mixture was taken at different initial diosgenin concentrations, and 1.9 mL of the ABTS· radical solution was added [22]. The percentage of inhibition of the ABTS· radical for the extract mixtures was evaluated using Equation (1) [71]:

%Inhibition = ((A_0_ − A7)A_0_) × 100 (1)

where A_0_ is the absorbance of the ABTS· radical at time zero, and A7 is the absorbance of the ABTS· radical after thirty minutes of incubation.

For the DPPH· method, the methodology proposed by Brand-Williams et al. [72] was followed with certain modifications. A solution of DPPH· in methanol (25 mg L^−1^) was prepared, 3.9 mL of the methanol solution was taken, and 0.1 mL of the extract mixture was added to different initial concentrations of diosgenin. Then, it was mixed vigorously and incubated for 30 min in the dark at room temperature. Absorbance was measured at 517 nm with a UV–Visible spectrophotometer (Shimadzu, Kyoto, Japan). Measurements were made in triplicate, using methanol as a blank and a diosgenin solution as a control. The percentage inhibition of the DPPH· radical was calculated for the ABTS· radical, for the extracts obtained from the bioconversion process, and for diosgenin.

### 4.7. Determination of Antifungal Activity

Antifungal activity was performed in PDA medium in Petri dishes in triplicate. Once solidified, with the help of a mold, conical-shaped wells were formed in the agar with a maximum volume of 50 μL; 20 μL of extract from the bioconversion process was added to each well and left to dry for 2 h. After 2 h, a cell suspension of 150 microliters equivalent to 1 × 10^6^ cell mL^−1^ was inoculated. Subsequently, they were incubated at 30 °C, and the inhibition was evaluated for 8 days. The well diffusion method was chosen for its ability to provide clear, quantifiable results regarding the inhibition of fungal hyphal growth. Although this method may not be the traditional choice for all fungal assays, it has been successfully used in previous studies to assess antifungal activity in a controlled environment [73]. The well diffusion method allowed us to monitor the fungi’s radial growth and assess the bioconverted extracts’ inhibitory effects in a reproducible and reliable manner. The antifungal activity was determined by estimating the radial growth area of the *Botrytis cinerea*, *Alternaria* sp., and *Aspergillus niger* strains for all the extracts from the diosgenin bioconversion process. The inhibition halo of the fungus growth was estimated at 24, 48, 96, 144, and 192 h of incubation with the help of a vernier. Finally, the inhibition (%) of the growth of the fungus was calculated with Equation (2). The measurements were carried out in triplicate, and the strain was used as a control without bioconversion extracts.

%Inhibition = (DB − DTDB) × 100 (2)

DB is the diameter of the corresponding blank (fungus without bioconversion extracts), and DT is the diameter in mm of the related treatment (fungus with bioconversion extracts).

### 4.8. Determination of Herbicidal Activity

The herbicidal activity was determined in a climatic chamber under controlled conditions of humidity (80%), temperature (25 ± 2.5 °C), and a photoperiod of 12 h of light and 12 h of darkness. The experimental units comprised Petri dishes and filter paper discs sterilized and moistened with distilled water. The model seeds were species of crops belonging to the monocotyledonous and dicotyledonous groups. Each treatment condition involved planting 9 seeds per replicate, with three replicates per treatment group, totaling 27 seeds per treatment. The bioconverted diosgenin extracts were applied by imbibition, ensuring uniform coverage across all seeds. The treatments were administered at the same growth stage to maintain consistency. The inhibition (%) of the growth of the seedlings was calculated with Equation (3). The measurements were carried out in triplicate, and an herbicidal (Picloram+2,4-Duzzi) was used as a control.

%Inhibition = ((SP − SG)/SP) × 100 (3)

where SP is the seeds planted, and SG is the seeds germinated.

### 4.9. Statistical Analysis

The statistical analysis of antioxidant, antifungal, and herbicidal activity was conducted using SigmaPlot v11 (Systat Software Inc., Chicago, IL, USA). An ANOVA was performed (*p* < 0.05), followed by the Holm-Sidak post-hoc test (*p* < 0.05). Each experiment was conducted with three independent biological replicates, and each biological replicate was performed in duplicate, resulting in six data points per treatment condition. This design is consistent with standard practices in biological assays. Statistical analyses were performed to confirm the reproducibility and reliability of the results.

## 5. Conclusions

This study demonstrated the successful bioconversion of diosgenin by the marine yeast *Yarrowia lipolytica P01a*, producing bioactive compounds protodioscin and soyasaponin I. The bioconversion process involved glycosylation and hydroxylation reactions, enhancing the resulting extracts’ antioxidant, antifungal, and herbicidal activities.

The bioconversion process varied significantly with different initial concentrations of diosgenin, with optimal yields at specific concentrations. Notably, protodioscin and soya-saponin I were produced, demonstrating the effectiveness of *Y. lipolytica P01a* in transforming diosgenin into these valuable saponins. The bioconverted extracts exhibited significantly higher antioxidant activity compared to the diosgenin precursor. The highest antioxidant activity was observed with 1000 mg L^−1^ of initial diosgenin, showing 97.02% and 33.30% inhibition of ABTS· and DPPH· radicals, respectively. The extracts showed potent antifungal effects, particularly against *Botrytis cinerea*, with notable inhibition of radial growth at various concentrations of diosgenin. This result suggests the potential of these bioconverted saponins as natural antifungal agents. The diosgenin extracts from the bioconversion process demonstrated effective herbicidal activity on different model seed species, comparable to commercial herbicides. This fact highlights their potential as eco-friendly alternatives for weed management in agriculture.

Overall, the study presents a promising biotechnological approach for producing natural biofungicides and bioherbicides, contributing to safer and more sustainable agricultural practices.

## Figures and Tables

**Table 1:** Saponin Concentrations from Diosgenin Bioconversion by *Yarrowia lipolytica P01a*. It shows the concentrations (mg/L) of saponins produced by *Yarrowia lipolytica* P01A during the bioconversion of diosgenin. The results illustrate the microbial efficiency in converting diosgenin into bioactive saponins, such as protodioscin and soyasaponin I, highlighting the potential applications in pharmaceutical and agricultural industries. The yield (Y) shows the relation between product (P) and substrate (S). ND = Non detectable.

Initial Concentration of Diosgenin (mg L^−1^)								
	10	20	30	40	50	60	70	80–1000
Protodioscin (mg L^−1^)	4.25 ± 0.00	16.53 ± 2.50	3.45 ± 0.01	5.31 ± 0.21	2.87 ± 0.01	ND	ND	ND
Soyasaponin I (mg L^−1^)	3.47 ± 0.00	2.86 ± 0.00	1.75 ± 0.00	2.94 ± 0.00	10.97 ± 0.59	7.70 ± 0.01	18.04 ± 0.01	ND
YP/S	0.772	0.969	0.173	0.206	0.276	0.128	0.257	ND

**Table 2:** Herbicidal Activity of Bioconverted Diosgenin Extracts on Monocotyledonous and Dicotyledonous Seeds. This table summarizes the herbicidal effects of diosgenin extracts obtained from the bioconversion process. The data show the percentage inhibition of seed germination in monocotyledonous (*Zea mays* L., *Triticum aestivum*, and *Hordeum vulgare*) and dicotyledonous (*Phaseolus vulgaris*) species. The extracts were tested at various concentrations, demonstrating significant herbicidal activity. The effectiveness of the bioconverted extracts suggests their potential use as natural herbicides in agricultural settings. The controls used were the herbicide Picloram+2,4-Duzzi and *Yarrowia lipolytica* P01a crude extract (control not included in the table because it did not affect seeds).

Initial Concentration of Diosgenin (mg L^−1^)	Corn			Wheat			Barley			Bean		
	% Inhibition											
	Diosgenin Extract	Diosgenin	Herbicide (Picloram)+2,4-D)	Diosgenin Extract	Diosgenin	Herbicide (Picloram+2,4-D)	Diosgenin Extract	Diosgenin	Herbicide (Picloram+2,4-D)	Diosgenin Extract	Diosgenin	Herbicide (Picloram+2,4-D)
10	100 ± 0.00	68.88 ± 3.25	100 ± 0.00	100 ± 0.00	86.66 ± 0.00	100 ± 0.00	100 ± 0.00	87.11 ± 1.72	100 ± 0.00	100 ± 0.00	92.44 ± 2.34	100 ± 0.00
20	100 ± 0.00	79.55 ± 1.72	100 ± 0.00	100 ± 0.00	93.33 ± 0.00	100 ± 0.00	100 ± 0.00	92.44 ± 2.34	100 ± 0.00	100 ± 0.00	93.33 ± 0.00	100 ± 0.00
30	100 ± 0.00	72.00 ± 2.76	100 ± 0.00	100 ± 0.00	88.88 ± 3.25	100 ± 0.00	100 ± 0.00	92.00 ± 2.76	100 ± 0.00	100 ± 0.00	100 ± 0.00	100 ± 0.00
40	100 ± 0.00	100 ± 0.00	100 ± 0.00	100 ± 0.00	100 ± 0.00	100 ± 0.00	100 ± 0.00	100 ± 0.00	100 ± 0.00	100 ± 0.00	100 ± 0.00	100 ± 0.00
50	100 ± 0.00	100 ± 0.00	100 ± 0.00	100 ± 0.00	100 ± 0.00	100 ± 0.00	100 ± 0.00	100 ± 0.00	100 ± 0.00	100 ± 0.00	100 ± 0.00	100 ± 0.00
60	100 ± 0.00	100 ± 0.00	100 ± 0.00	100 ± 0.00	100 ± 0.00	100 ± 0.00	100 ± 0.00	100 ± 0.00	100 ± 0.00	100 ± 0.00	100 ± 0.00	100 ± 0.00
70	100 ± 0.00	100 ± 0.00	100 ± 0.00	100 ± 0.00	100 ± 0.00	100 ± 0.00	100 ± 0.00	100 ± 0.00	100 ± 0.00	100 ± 0.00	100 ± 0.00	100 ± 0.00
80	100 ± 0.00	100 ± 0.00	100 ± 0.00	100 ± 0.00	100 ± 0.00	100 ± 0.00	100 ± 0.00	100 ± 0.00	100 ± 0.00	100 ± 0.00	100 ± 0.00	100 ± 0.00
90	100 ± 0.00	100 ± 0.00	100 ± 0.00	100 ± 0.00	100 ± 0.00	100 ± 0.00	100 ± 0.00	100 ± 0.00	100 ± 0.00	100 ± 0.00	100 ± 0.00	100 ± 0.00
100	100 ± 0.00	100 ± 0.00	100 ± 0.00	100 ± 0.00	100 ± 0.00	100 ± 0.00	100 ± 0.00	100 ± 0.00	100 ± 0.00	100 ± 0.00	100 ± 0.00	100 ± 0.00
200	100 ± 0.00	100 ± 0.00	100 ± 0.00	100 ± 0.00	100 ± 0.00	100 ± 0.00	100 ± 0.00	100 ± 0.00	100 ± 0.00	100 ± 0.00	100 ± 0.00	100 ± 0.00
400	100 ± 0.00	100 ± 0.00	100 ± 0.00	100 ± 0.00	100 ± 0.00	100 ± 0.00	100 ± 0.00	100 ± 0.00	100 ± 0.00	100 ± 0.00	100 ± 0.00	100 ± 0.00
600	100 ± 0.00	100 ± 0.00	100 ± 0.00	100 ± 0.00	100 ± 0.00	100 ± 0.00	100 ± 0.00	100 ± 0.00	100 ± 0.00	100 ± 0.00	100 ± 0.00	100 ± 0.00
800	100 ± 0.00	100 ± 0.00	100 ± 0.00	100 ± 0.00	100 ± 0.00	100 ± 0.00	100 ± 0.00	100 ± 0.00	100 ± 0.00	100 ± 0.00	100 ± 0.00	100 ± 0.00
1000	100 ± 0.00	100 ± 0.00	100 ± 0.00	100 ± 0.00	100 ± 0.00	100 ± 0.00	100 ± 0.00	100 ± 0.00	100 ± 0.00	100 ± 0.00	100 ± 0.00	100 ± 0.00

**Table 3:** Comparison of Saponin Production Through Various Bioconversion Processes. This table provides a comparative overview of saponin production through various bioconversion processes, summarizing the microbial systems used, initial substrates, main saponins produced, saponin yields, and the resulting enhancements in bioactivity. The table demonstrates that the method used in this study yields a significant amount of bioactive saponins, particularly with enhanced antioxidant, antifungal, and herbicidal properties, comparable or superior to other methods.

Study	Microbial System	Initial Substrate	Metabolites Produced	Saponin Yield (mg/L)	Enhanced Bioactivity
This study	*Yarrowia lipolytica P01a*	Diosgenin	Protodioscin, Soyasaponin I	Up to 19.39	Significant increase in antioxidant, antifungal, and herbicidal activities
Dong et al., 2015 [36]	*Penicillium dioscin*	Dioscin	Diosgenin	>90% conversion yield	High bioactivity, precursor for steroidal hormones
Liu et al., 2010 [37]	*Trichoderma harzianum*	Steroidal saponins	Diosgenin	30.05 mg/g	High yield via optimized biotransformation; potential for eco-friendly diosgenin production.
Wu et al., 2011 [38]	*Coriolus versicolor*	Diosgenin	Diosgenin Derivatives	25 mg from compound **5** 8 mg from compound **8**	Novel hydroxylated diosgenin derivatives, indicating new pathways for steroidal modifications.
Dong et al., 2010 [39]	*Cunninghamella echinulata CGMCC*	Diosgenin	Diosgenin derivatives	20 mg from derivative 2 12 mg from compound **3** 80 mg from compound **4** 100 mg from compound **5**	Hydroxylation products with no significant cytotoxicity; potential for further pharmaceutical applications.
Xiao et al., 2011 [40]	*Cunninghamella echinulata CGMCC*	Diosgenin	Diosgenin derivatives	Not specified	Hydroxylation products contributing to novel steroidal structures with potential bioactivities.
Wang et al., 2007 [41]	*Streptomyces virginiae IBL-14*	Diosgenin, Diosgenone	Isonuatigenone, 25β-hydroxy derivatives	28.4% of Diosgenin bioconversion	Pathway elucidation for converting diosgenin to isonuatigenone suggests potential drug synthesis.

## Data Availability

The data presented in this study are available on request from the corresponding author.

## Supplementary Materials

The following supporting information can be downloaded at https://www.mdpi.com/article/10.3390/plants13182629/s1 Table S1. Radial growth of *Botrytis cinerea* treated with (a) diosgenin bioconversion extract (70 mg/L), (b) diosgenin, and (c) *Y. lipolytica P01a* extract. Table S2. Radial growth of *Alternaria sp*. treated with (a) diosgenin bioconversion extract (800 mg/L), (b) diosgenin, and (c) *Y. lipolytica P01a* extract. Table S3. Radial growth of *Aspergillus niger* treated with (a) diosgenin bioconversion extract (100 mg/L), (b) diosgenin, and (c) *Y. lipolytica P01a* extract. Figure S1. Herbicidal activity in corn treated with (a) diosgenin bioconversion extract, (b) diosgenin, (c) commercial herbicide, and (d) *Y. lipolytica P01a* extract. Figure S2. Herbicidal activity in beans treated with (a) diosgenin bioconversion extract, (b) diosgenin, (c) commercial herbicide, and (d) *Y. lipolytica P01a* extract.
