# Supplementary material for: Enhanced Antioxidant, Antifungal, and Herbicidal Activities through Bioconversion of Diosgenin by Yarrowia lipolytica P01a
Source: Plants (Basel). 2024 Sep 20;13(18):2629. doi: 10.3390/plants13182629 (PMC11434977; doi:10.3390/plants13182629)
Supplement: Supplementary file 1 [file plants-13-02629-s001.zip › plants-3148691-supplementary.pdf]

Supplementary Data

Table S1. Radial growth of *Botrytis cinerea* treated with a) diosgenin bioconversion extract (70 mg/L), b) diosgenin, and c) Y. *lipolytica P01a* extract.

Extracts	1 Days	2 Days	4 Days	6 Days	8 Days
a) Diosgenin bioconversion extract at 70 mg L^{-1}	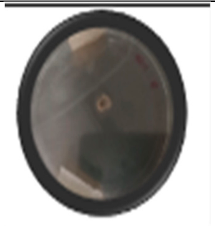	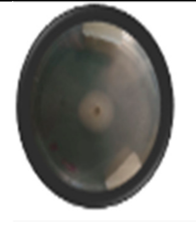	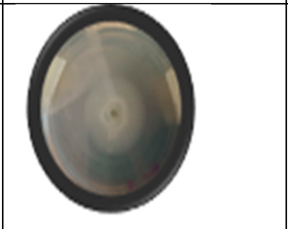	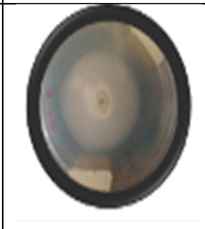	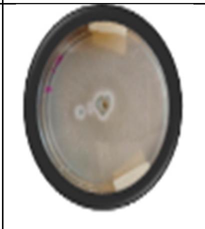
b) Diosgenin	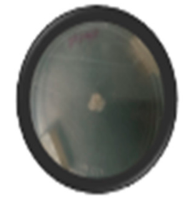	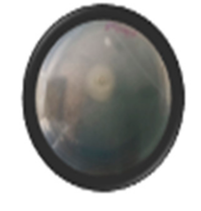	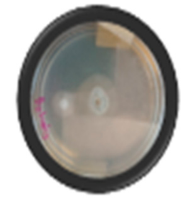	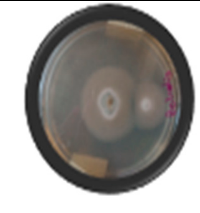	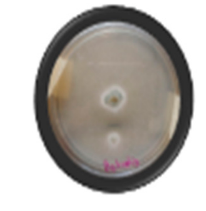
c) Extract from *Y. lypolitica* P01a	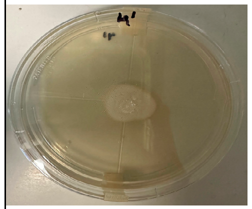	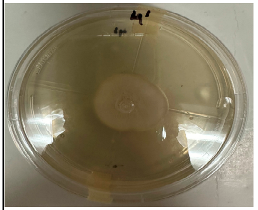	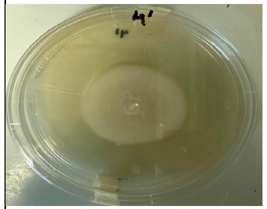	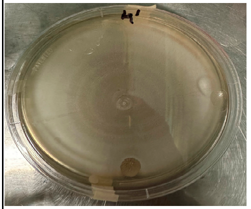	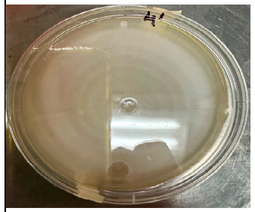

Table S2. Radial growth of *Alternaria sp.* treated with a) diosgenin bioconversion extract (800 mg/L), b) diosgenin, and c) *Y. lipolytica* P01a extract.

Extracts	1 Days	2 Days	4 Days	6 Days	8 Days
a) Diosgenin bioconversion extract at 800 mg L^{-1}	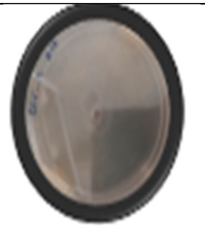	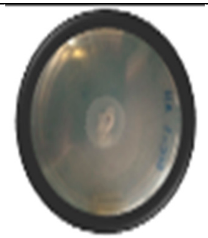	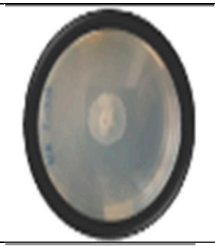	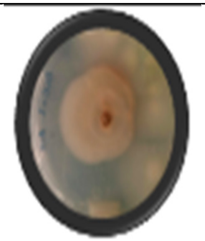	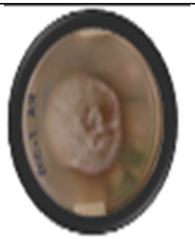
b) Diosgenin	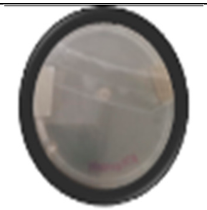	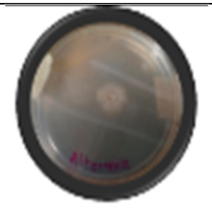	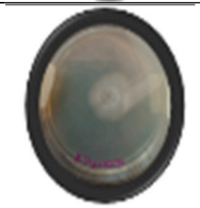	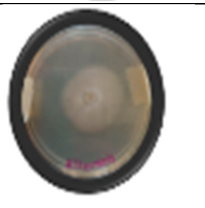	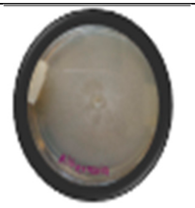
c) Extract from *Y. lypolitica* P01a	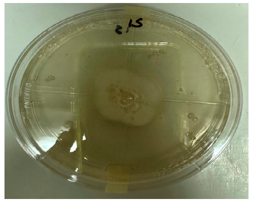	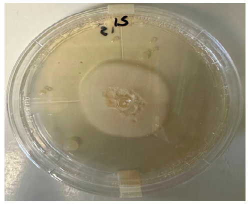	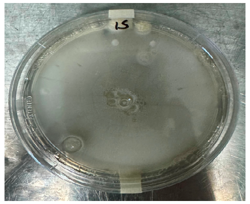	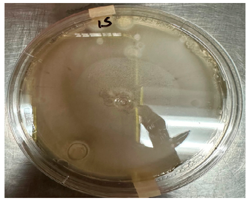	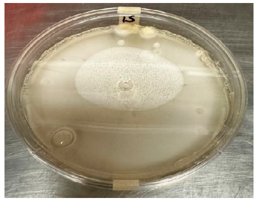

Table S3. Radial growth of *Aspergillus niger* treated with a) diosgenin bioconversion extract (100 mg/L), b) diosgenin, and c) *Y. lipolytica P01a* extract.

Extracts	1 Days	2 Days	4 Days	6 Days	8 Days
a) Diosgenin bioconversion extract at 100 mg L^{-1}	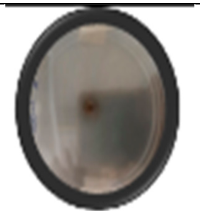	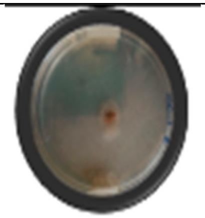	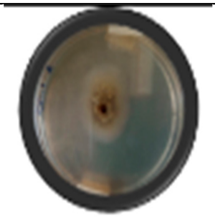	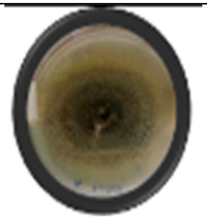	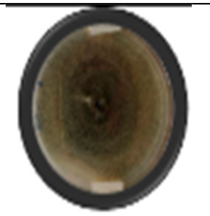
b) Diosgenin	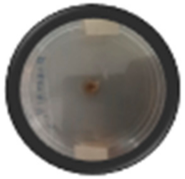	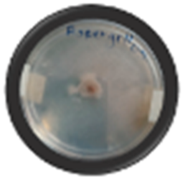	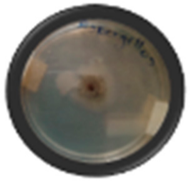	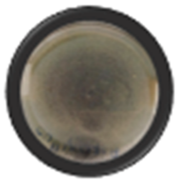	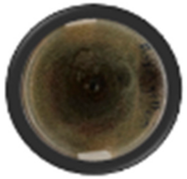
c) Extract from *Y. lypolitica* P01a	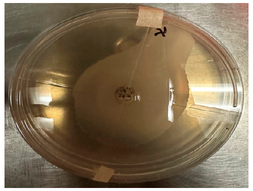	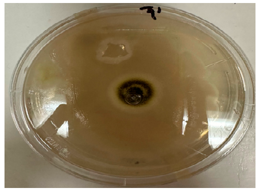	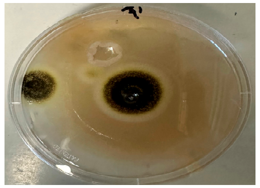	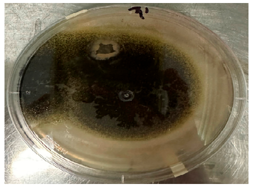	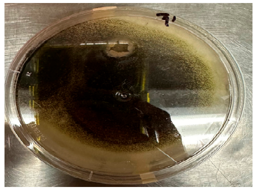

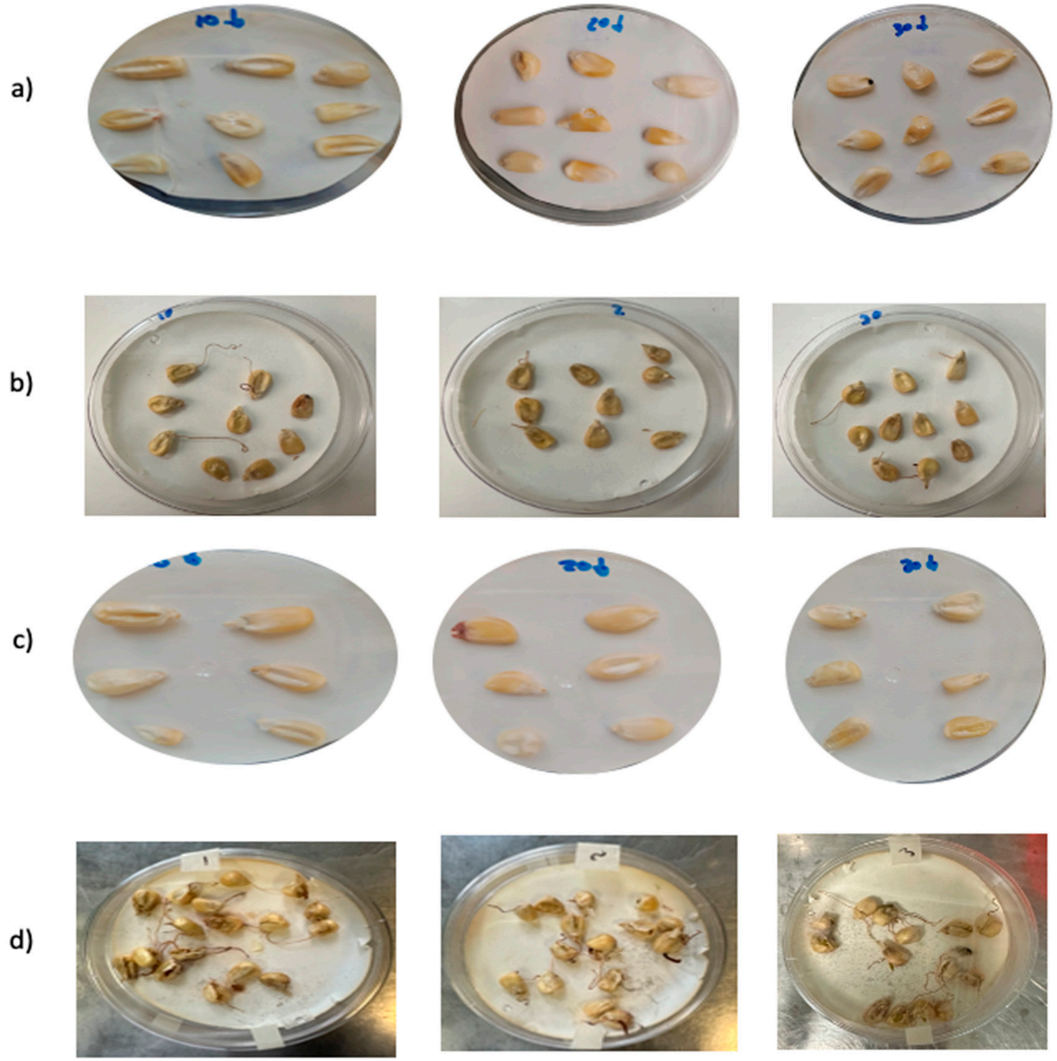

Figure S1. Herbicidal activity in corn treated with a) diosgenin bioconversion extract, b) diosgenin, c) commercial herbicide, and d) *Y. lipolytica P01a* extract.

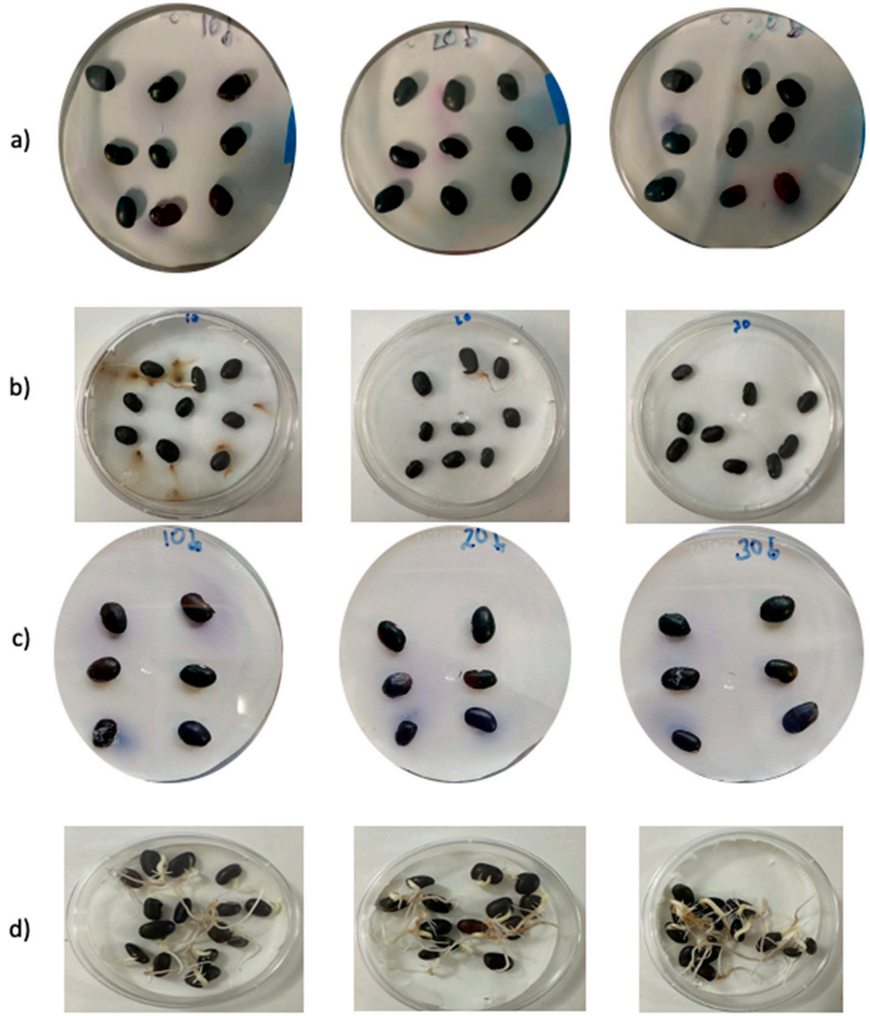

Figure S2. Herbicidal activity in beans treated with a) diosgenin bioconversion extract, b) diosgenin, c) commercial herbicide, and d) *Y. lipolytica P01a* extract.
